# Supplementary material for: Gender Moderates the Associations Between Responsiveness to Alarming Oral Sensations, Depressive Symptoms, and Dietary Habits in Adolescents
Source: Nutrients. 2025 May 12;17(10):1653. doi: 10.3390/nu17101653 (PMC12113894; doi:10.3390/nu17101653)
Supplement: Supplementary file 1 [file nutrients-17-01653-s001.zip › nutrients-3617764-supplementary.pdf]

Gender moderates the associations between responsiveness to alarming oral sensations, depressive symptoms, and dietary habits in adolescents

Leonardo Menghi [1,2,*], Lara Fontana [3], Silvia Camarda [4], Isabella Endrizzi [3], Maria Pina Concas [5], Paolo Gasparini [4,5], and Flavia Gasperi [1,3,*]

[1] Center Agriculture Food Environment, University of Trento, Via Mach 1, San Michele all'Adige, 38098, Italy; leonardo.menghi@unitn.it (L.M.); flavia.gasperi@unitn.it (F.G.)
[2] Department of Green Technology, University of Southern Denmark, Campusvej 55, Odense, 5230, Denmark; leom@igt.sdu.dk (L.M.)
[3] Research and Innovation Centre, Edmund Mach Foundation, Via Mach 1, San Michele all'Adige, 38098, Italy; lara.fontana@fmach.it (L.F.); isabella.endrizzi@fmach.it (I.E.); flavia.gasperi@fmach.it (F.G.)
[4] Department of Medicine, Surgery and Health Sciences, University of Trieste, Strada di Fiume 447, Trieste, 34149, Italy; silvia.camarda@phd.units.it (S.C.)
[5] Institute for Maternal and Child Health, I.R.C.C.S. "Burlo Garofolo", Via dell'Istria, 65/1, Trieste, 34137, Italy; mariapina.concas@burlo.trieste.it (M.P.C.); paolo.gasparini@burlo.trieste.it (P.G.)

*Correspondence to:

Leonardo Menghi: leom@igt.sdu.dk
Address: Campusvej 55, Odense, 5230, Denmark

Flavia Gasperi: flavia.gasperi@unitn.it
Via Mach 1, San Michele all'Adige, 38098, Italy.

Supplementary Materials

Figures ... **3**
Figure S1 ... 3
Figure S2 ... 4
Figure S3 ... 5
Figure S4 ... 6
Figure S5 ... 7
Figure S6 ... 8
Figure S7 ... 9
Figure S8 ... 10
Figure S9 ... 11
Figure S10 ... 12
Tables ... **13**
Table S1 ... 13
Table S2 ... 14
Table S3 ... 15
Table S4 ... 16
Table S5 ... 17
Table S6 ... 18
Table S7 ... 19
Table S8 ... 20
Table S9 ... 22
References ... **23**

Figures

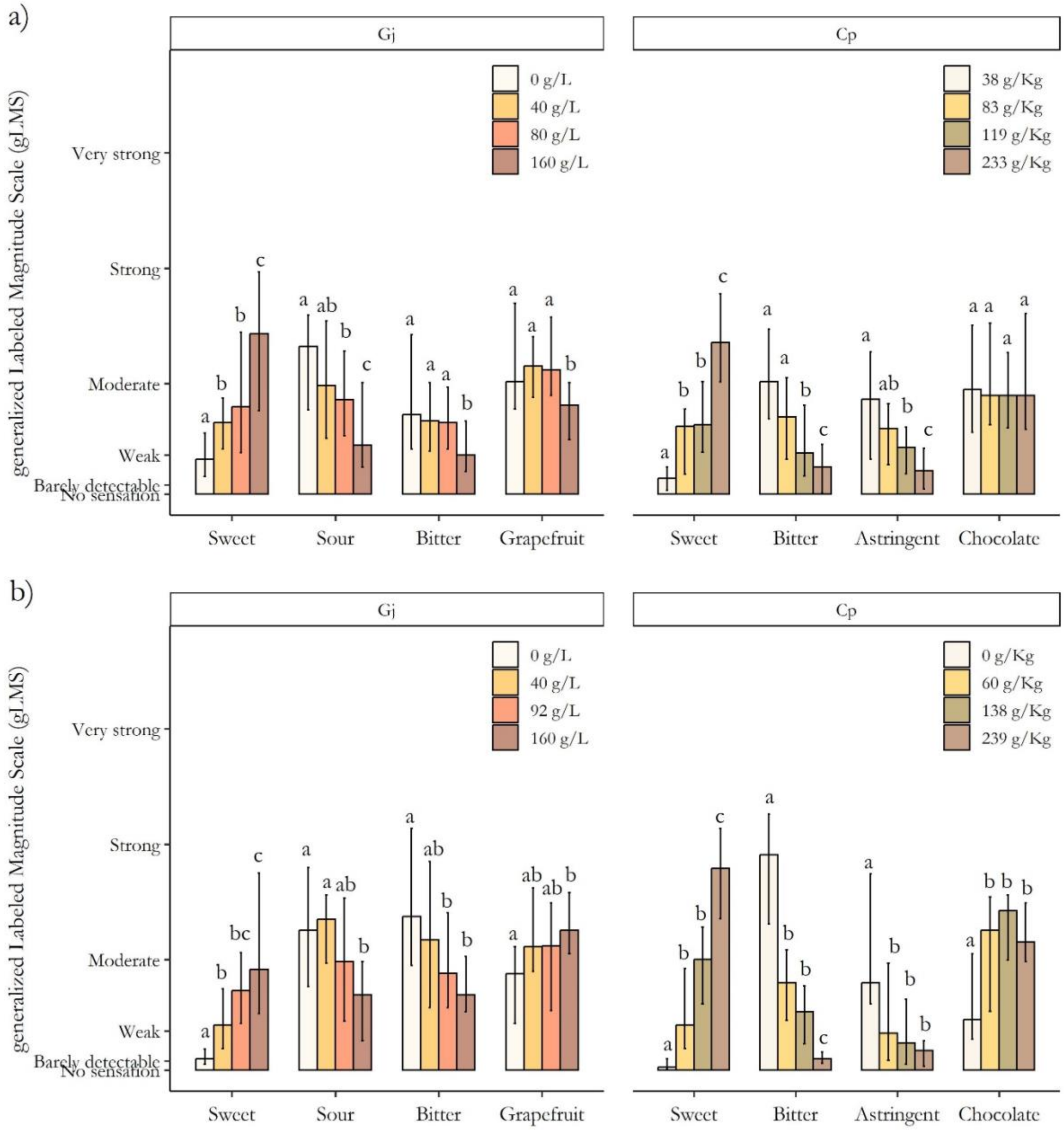

Figure S1: Sensory profiles of variants for each food model (Gj, Cp) as evaluated in Pilot 1 (n = 39) and Pilot 2 (n = 16). Samples were spiked with either previously tested (a) [1,2] or optimized (b) sucrose ranges (g/L; g/kg), respectively. Statistically significant differences between Gj and Cp variants for each sensory attribute (Sweet, Sour, Bitter, Astringent, Grapefruit, Chocolate) are indicated by different letters, based on Dunn's test with Bonferroni correction. Bars represent median ± IQR of the ratings.

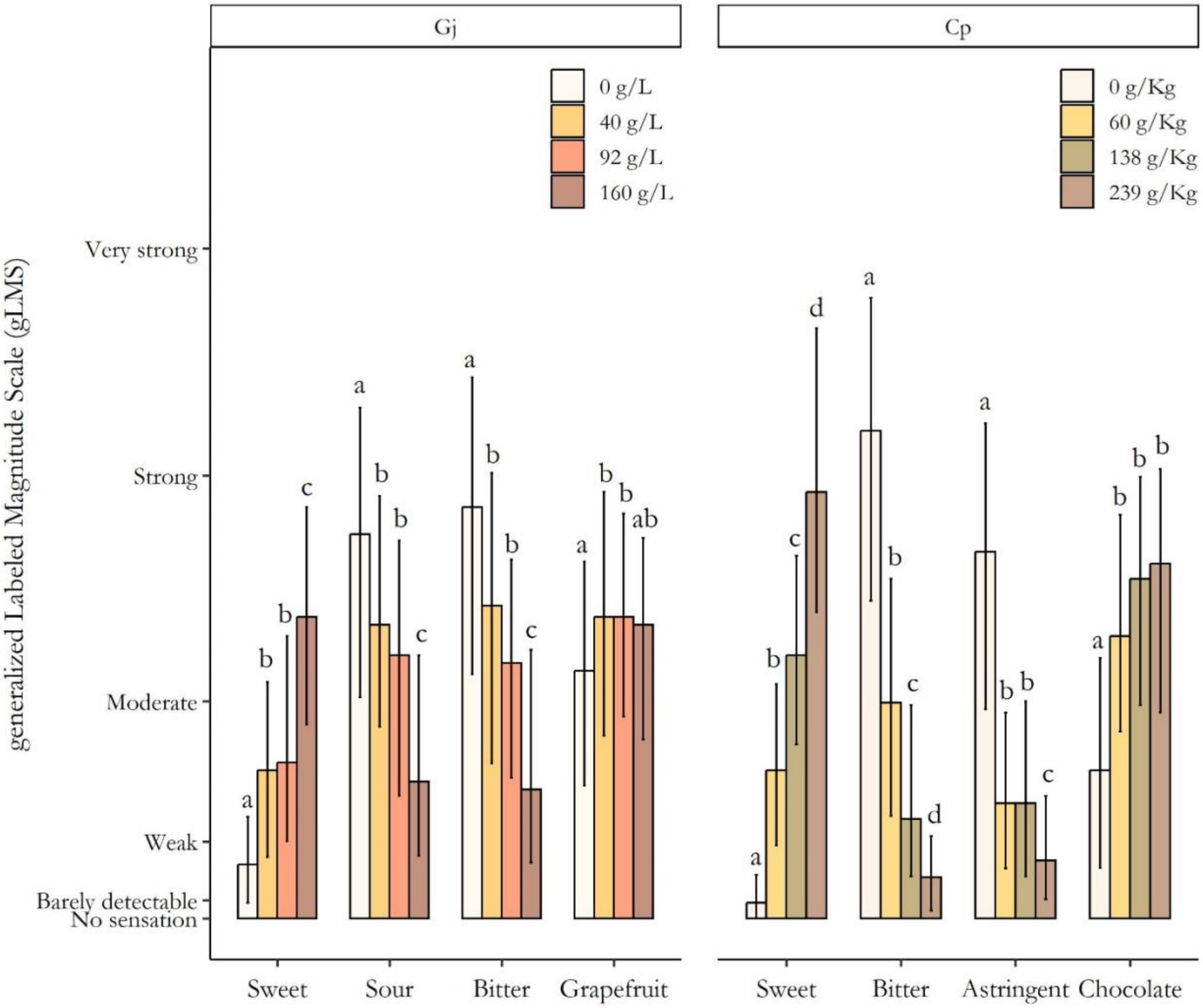

Figure S2: Variations in psychophysical responses (gLMS) to basic tastes (Sweet, Sour, Bitter), astringency, and flavors (Grapefruit, Chocolate) evoked by variants of grapefruit juice (Gj; n = 231) and dark chocolate pudding (Cp; n = 227) across increasing sucrose concentrations (g/L for Gj; g/kg for Cp). Statistically significant differences between Gj and Cp variants for each sensory attribute are indicated by different letters, based on Dunn's test with Bonferroni adjustment.

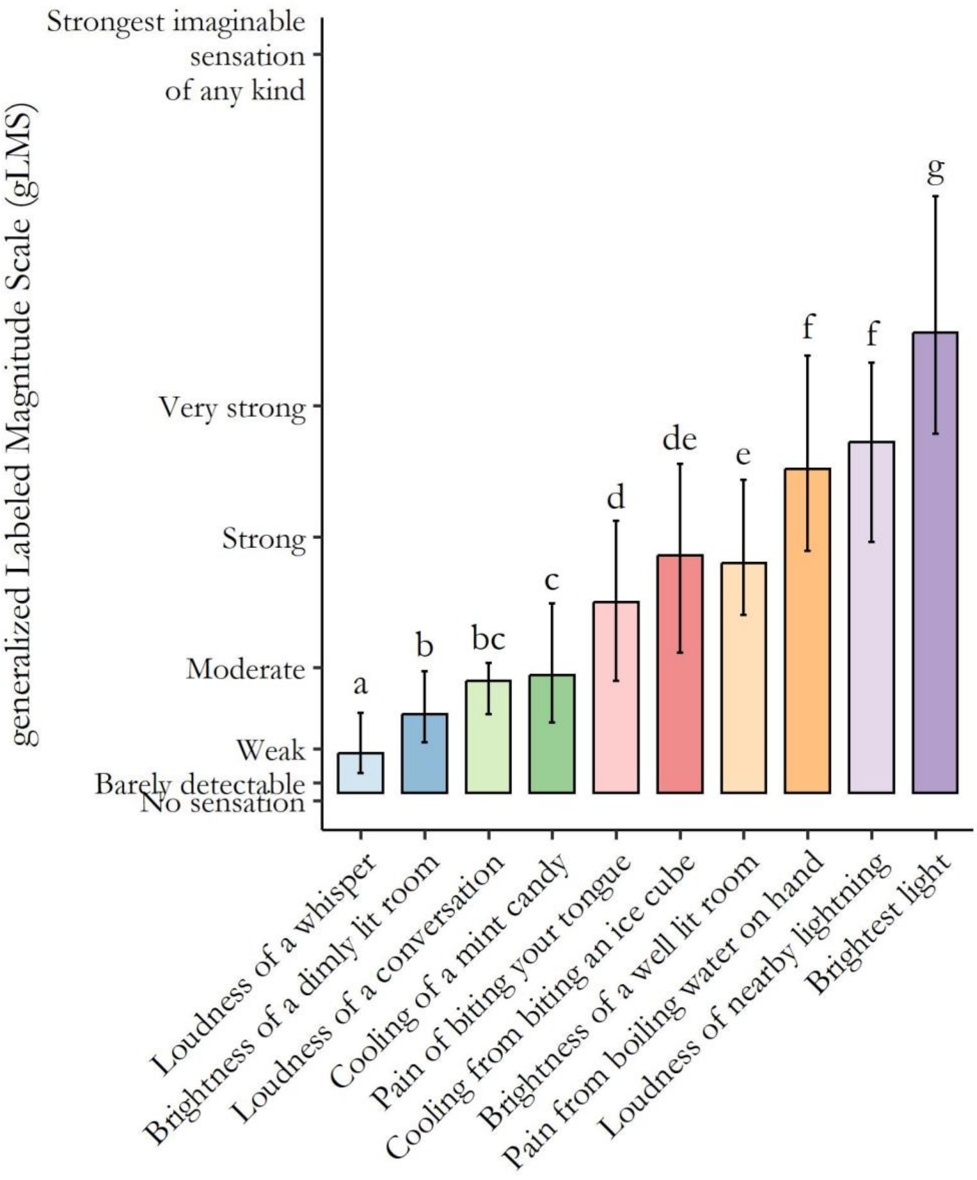

Figure S3: Differences in recalled intensities (gLMS) for the extraoral stimuli employed during the gLMS training [3]. Statistically significant pairwise differences ($p < 0.05$), identified using post hoc Dunn's test with Bonferroni correction, are indicated by different letters.

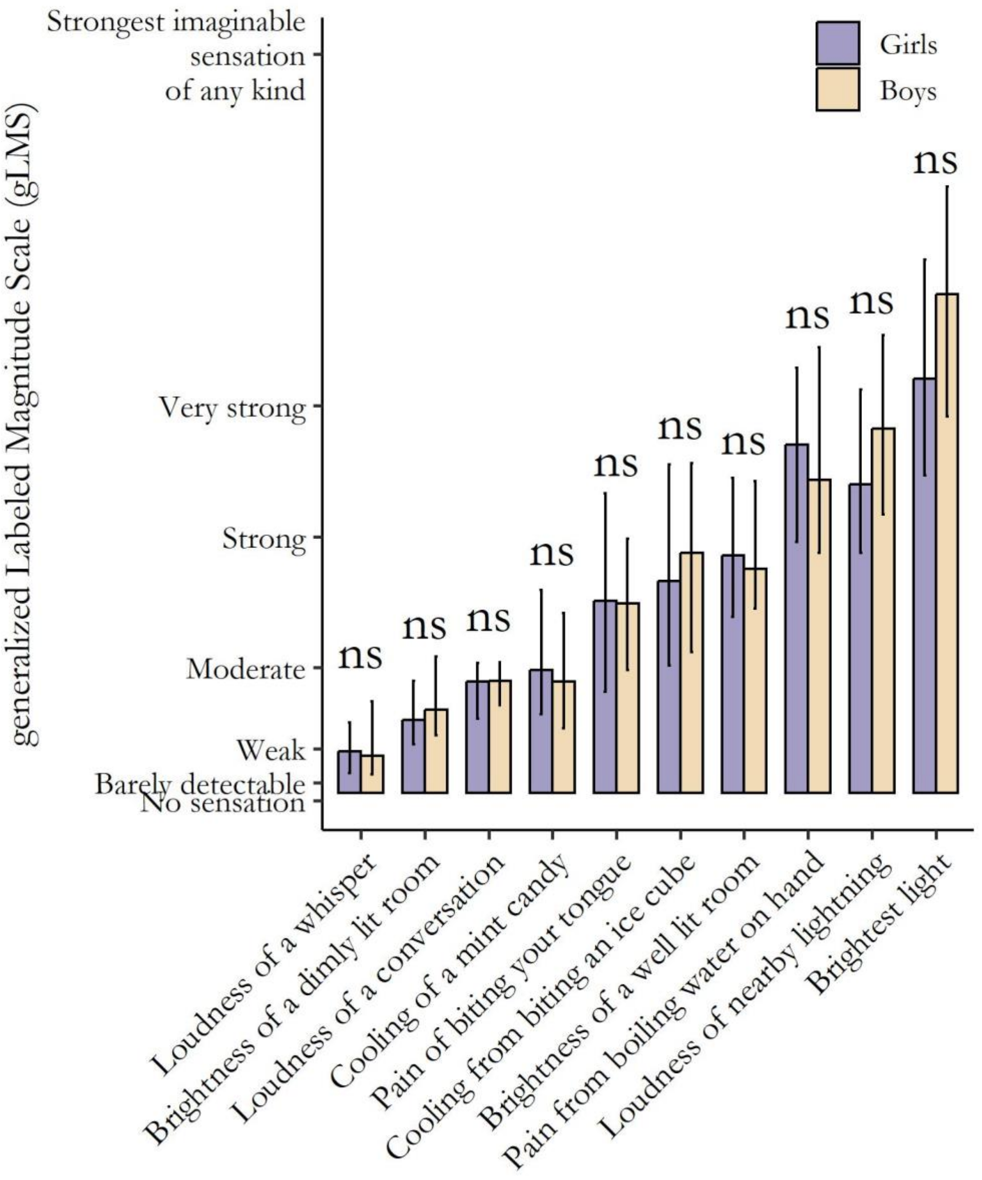

Figure S4: Girls and boys rated the ten extraoral stimuli employed during the gLMS training [3] as equally intense. Different letters indicate statistically significant pairwise differences ($p < 0.05$) identified via post hoc Dunn's test with Bonferroni adjustment.

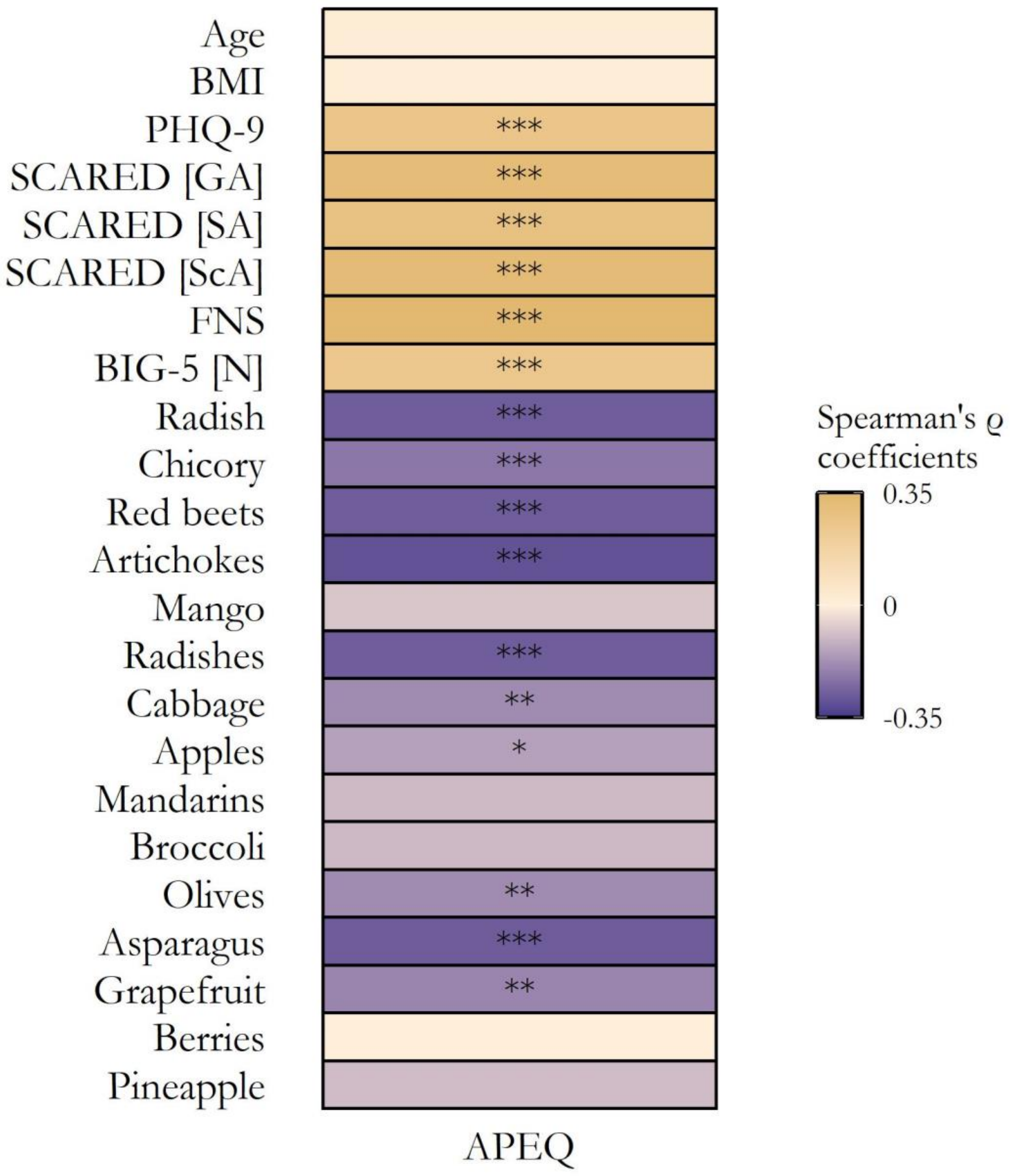

Figure S5: Correlations between the APEQ total score and both theoretically similar and dissimilar constructs (Spearman's ϱ coefficients). The APEQ total score exhibited adequate convergent validity, as evidenced by significant positive correlations with related states or traits such as food neophobia (FNS), depressive symptoms (PHQ-9), neuroticism (BIG-5 [N]), and generalized (SCARED [GA]), social (SCARED [SA]), and school (SCARED [ScA]) anxiety. Moreover, the discriminant validity of the APEQ total score was supported by significant inverse correlations with familiarity ratings (5-point Likert scale, 1 = "I do not recognize it"; 5 = "I regularly eat it") [2] for both vegetables and fruits e.g., [4–7]. * = $p < 0.05$; ** = $p < 0.01$; *** = $p < 0.001$.

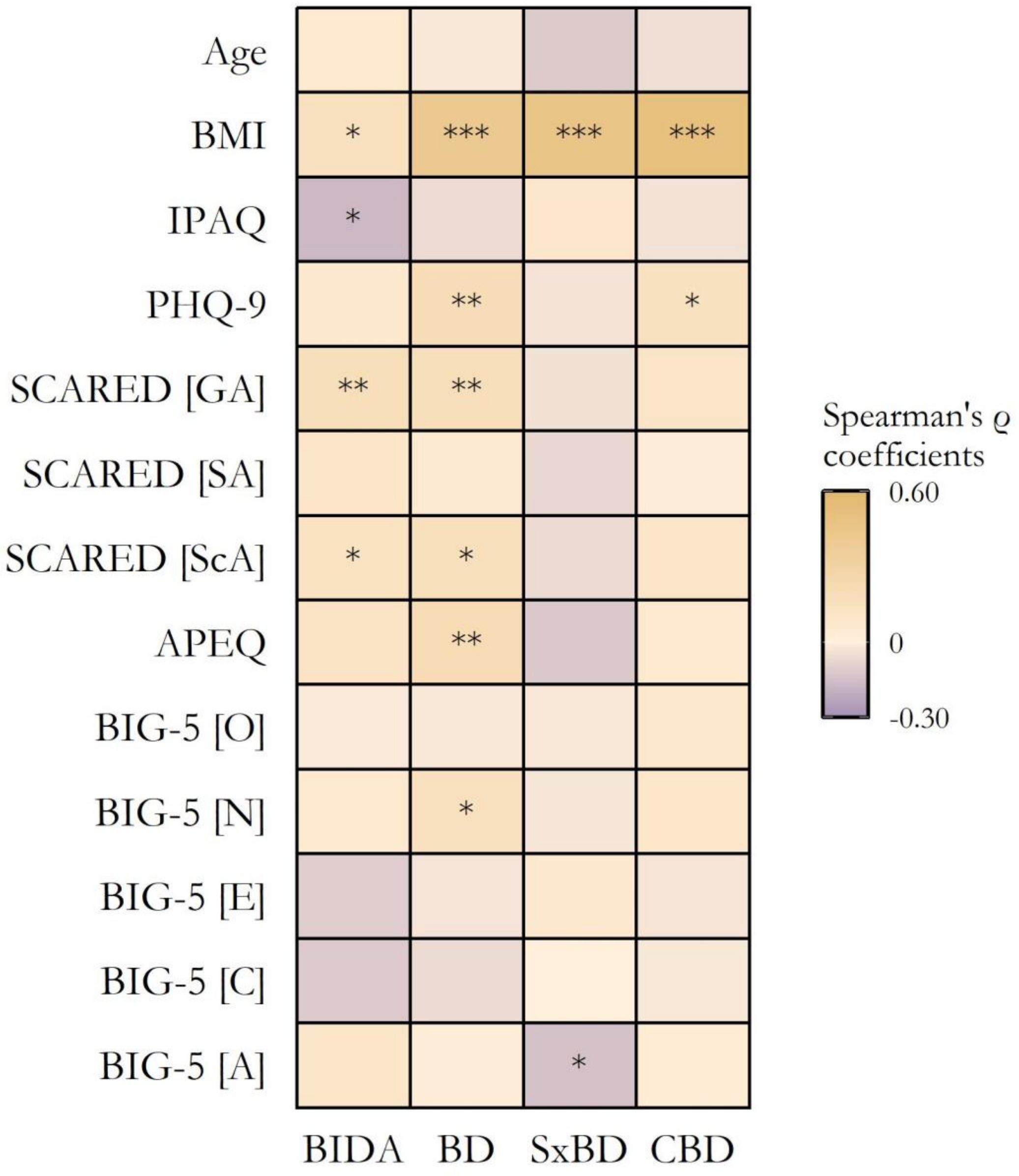

Figure S6: Correlations (Spearman's ϱ coefficients) between the BIDA total and subscale scores and theoretically similar and dissimilar factors. Initial evidence for the convergent and discriminant validity of the tool was demonstrated by: a) strong positive correlations between the BIDA scores and BMI [8], and between the BIDA and domains of anxiety (SCARED [GA]; SCARED [ScA]), depressive symptoms (PHQ-9), picky eating (APEQ) or neuroticism (BIG-5 [N]). Additionally, the inverse correlation between the BIDA composite score and engagement in physical activity (IPAQ) supported the discriminant validity of the measure [9]. * = $p < 0.05$; ** = $p < 0.01$; *** = $p < 0.001$.

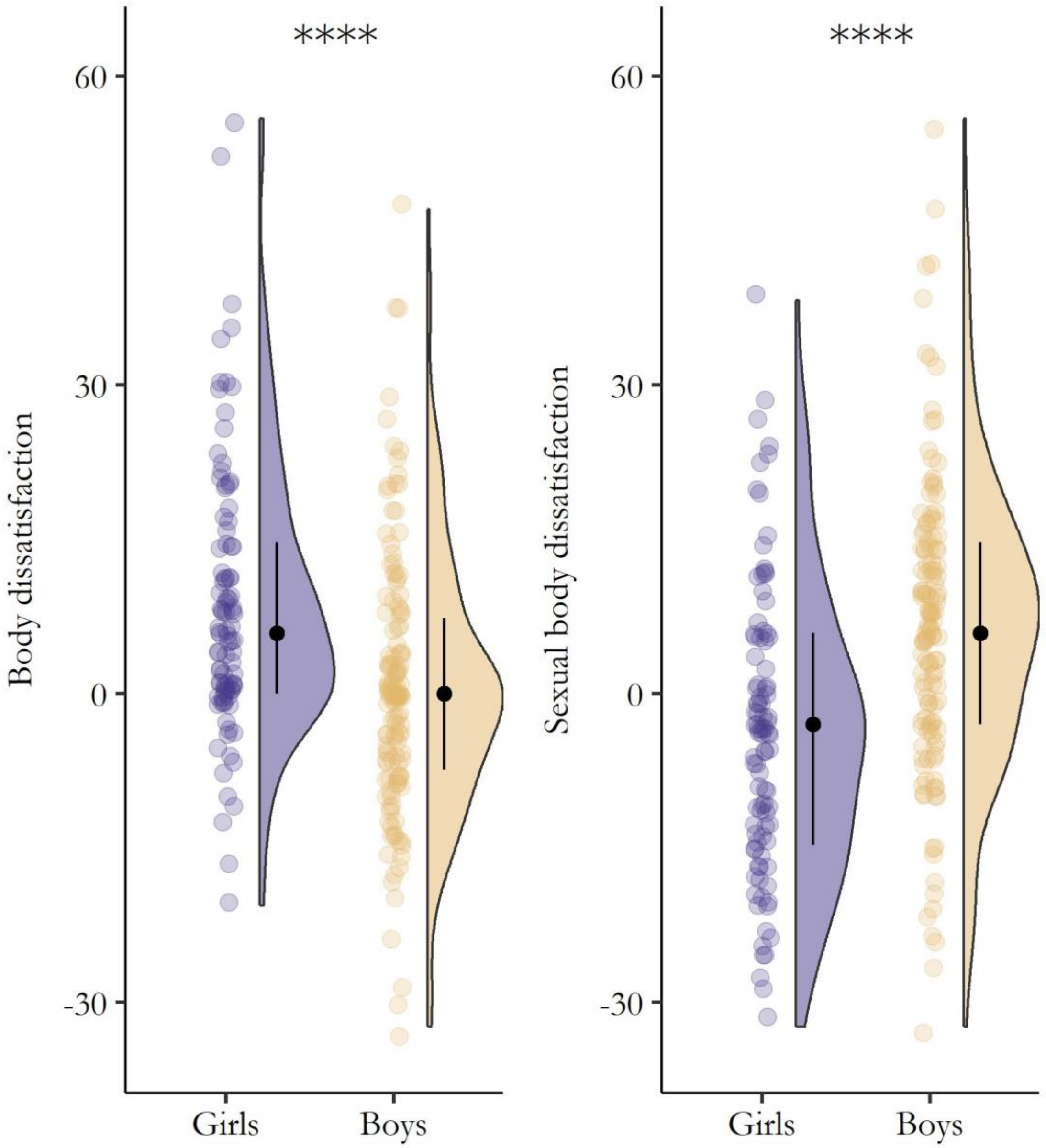

Figure S7: Differences (Wilcoxon Rank Sum Test) in body dissatisfaction (left) and sexual body dissatisfaction (right) between girls (slate blue) and boys (light tan). The plot displays raw data points (the "rain"), the kernel density estimate (the "cloud"), and the median (black filled circle) ± IQR (perpendicular black line). **** = $p < 0.0001$.

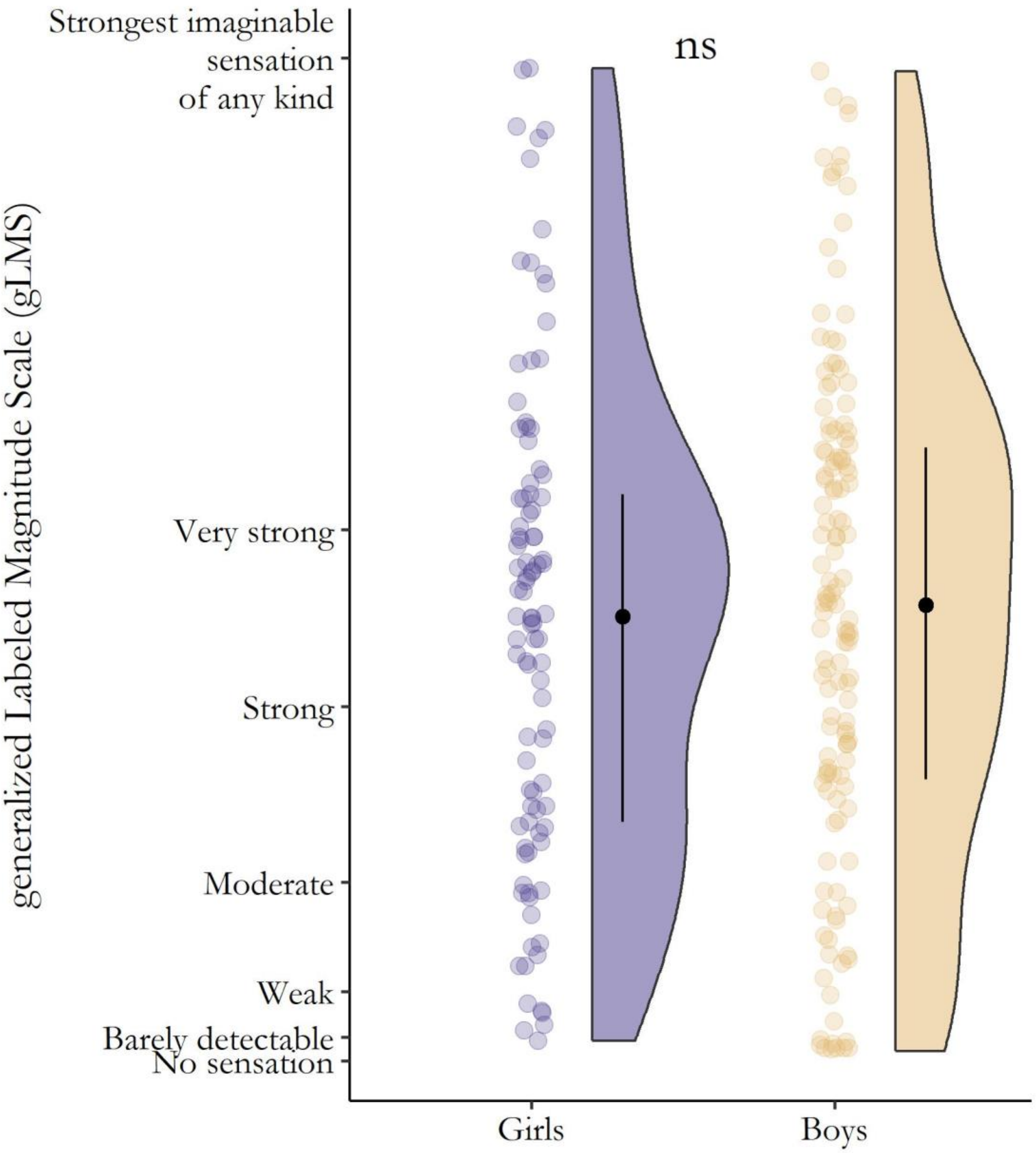

Figure S8: Variations in PROP responsiveness (gLMS) between genders (Wilcox Rank Sum Test). Raw observations (the rain), the estimated Kernel density (the cloud) and the median (black filled circle) ± IQR (black perpendicular line) are depicted. ns = $p > 0.05$.

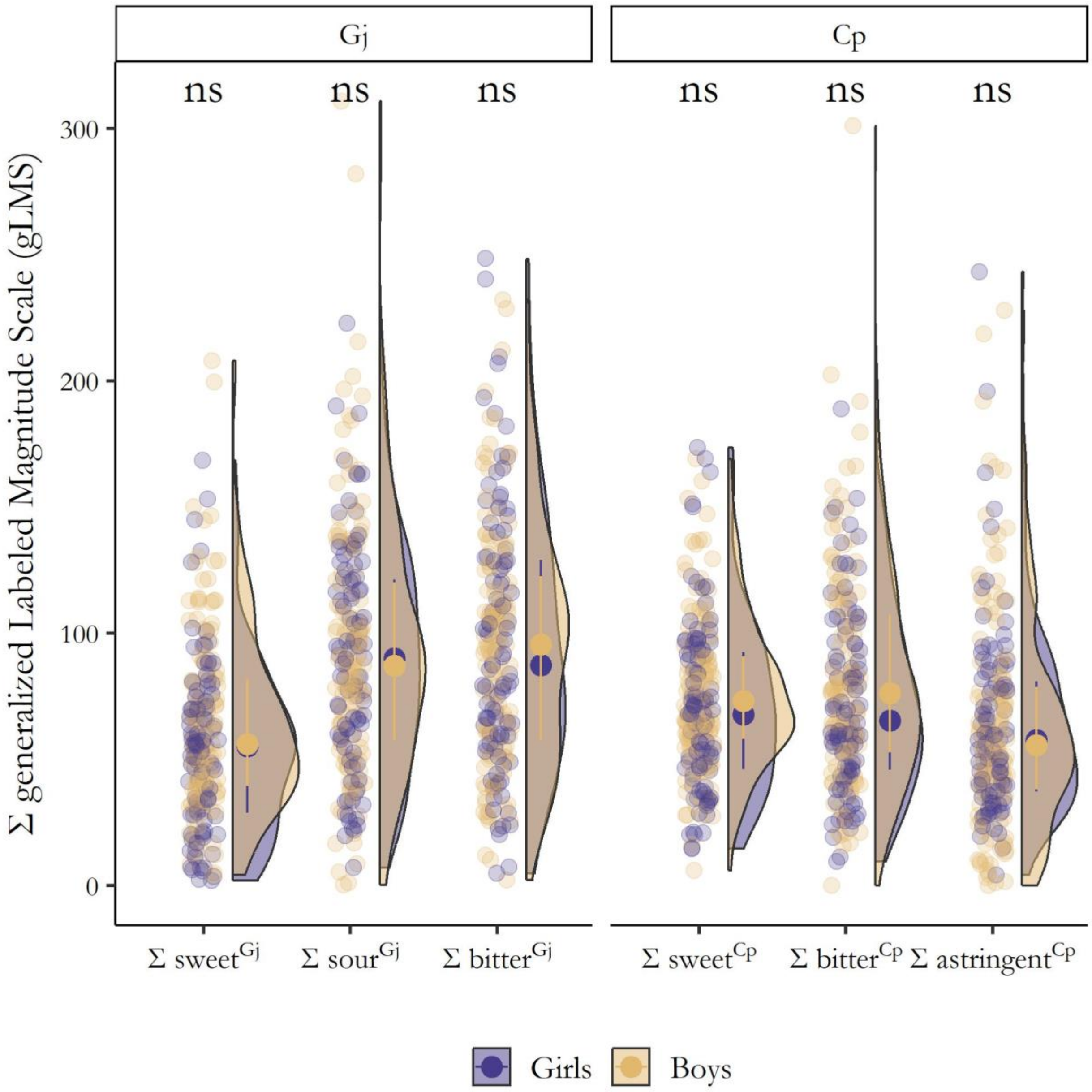

Figure S9: Differences in global (Σ) sensory acuity (gLMS) by gender (Dunn's test with Bonferroni adjustment). The figure presents raw observations (the "rain"), the estimated kernel density (the "cloud"), and the median (black filled circle) ± IQR (black perpendicular line). ns = $p > 0.05$. Gj: grapefruit juice; Cp: dark chocolate pudding.

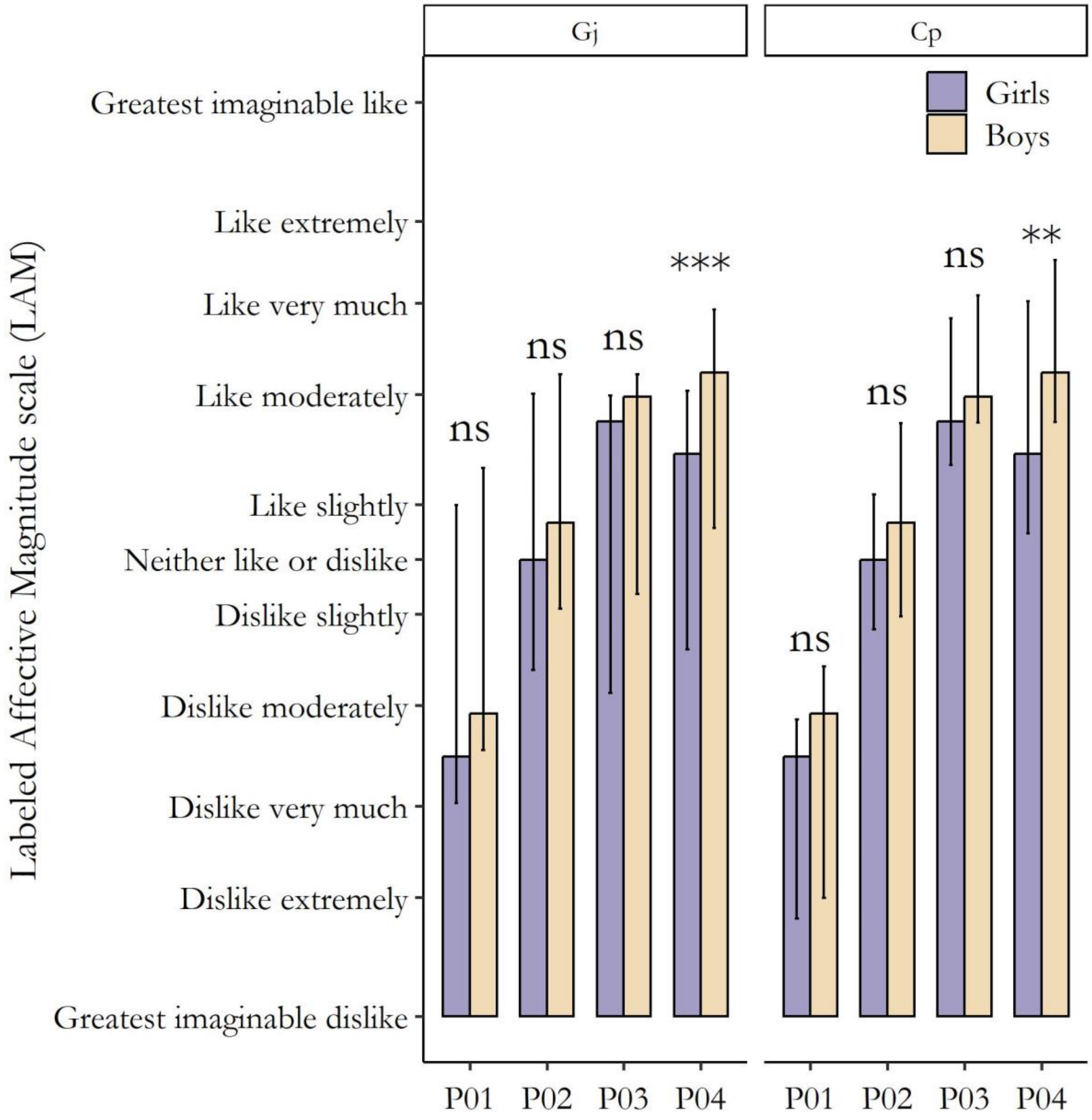

Figure S10: Differences between genders in liking (LAM) for all variants (P01-P04) of grapefruit juice (Gj) and dark chocolate pudding (Cp) according to Dunn's test with Bonferroni adjustment. Bars represent the medians ± IQR of the ratings. ns = $p > 0.05$; ** = $p < 0.01$; *** = $p < 0.001$.

Tables

	TOT (n = 232)	Girls (n = 97)	Boys (n = 135)	p.value
**Age***	14.5 ± 0.6	14.6 ± 0.7	14.5 ± 0.6	0.331†
**BMI**	21.0 ± 3.2	20.6 ± 2.7	21.3 ± 3.5	0.095†
**IPAQ**	4278 ± 3081	3703 ± 2617	4691 ± 3324	**0.012†**
**Diet****				
Omnivores	172	67	105	0.194††
Flexitarians	59	29	30	
Vegetarians	1	1	0	
**Smoking frequency per month**				
**Cigarettes**				
Never	209	82	127	
1-9 days	14	9	5	0.113††
10-19 days	5	3	2	
> 20 days	4	3	1	
**E-cig**				
Never	196	77	119	
1-9 days	26	16	10	0.115††
10-19 days	7	2	5	
> 20 days	3	2	1	
**Alcohol units (12 g) per week**				
0	77	32	45	
< 1	97	40	57	0.775††
1-2	39	15	24	
≥ 3	19	10	9	
**Food allergies**				
No	224	93	131	0.663††
Yes	8	4	4	

†p.value calculated via Welch's t-test; ††p.value calculated via chi-squared test.
*mean ± SD; **n

Table S1: Demographic (mean ± SD), lifestyle (n), and diet-related (n) characteristics of participants (n = 232). Differences between girls (n = 97) and boys (n = 135) are also listed (p.value). Values in bold are intended as statistically significant ($p < 0.05$). IPAQ: International Physical Activity Questionnaire [10].

	BD	SxBD	CBD
**BD**	/		
**SxBD**	0.536***	/	
**CBD**	0.696***	0.673***	/

Table S2: Inter-item correlations (Spearman's rho coefficients) between subscale scores of the Body Image Dimensional Assessment (BIDA) [8]. BD: Body Dissatisfaction; SxBD: Sexual Body Dissatisfaction; CBD: Compared Body Dissatisfaction. *** = $p < 0.001$.

	TOT (n = 218)	Girls (n = 93)	Boys (n = 125)	p.value
**Age***	14.5 ± 0.7	14.6 ± 0.7	14.5 ± 0.6	0.363†
**BMI**	20.9 ± 3.1	20.5 ± 2.7	21.2 ± 3.3	0.115†
**IPAQ**	4273 ± 3101	3687 ± 2637	4709 ± 3350	**0.013†**
**Diet****				
Omnivores	162	65	97	0.259††
Flexitarians	55	27	28	
Vegetarians	1	1	0	
**Smoking frequency per month**				
**Cigarettes**				
Never	199	80	119	0.125††
1-9 days	12	8	4	
10-19 days	3	2	1	
> 20 days	4	3	1	
**E-cig**				
Never	187	75	112	0.136††
1-9 days	23	15	8	
10-19 days	6	2	4	
> 20 days	2	1	1	
**Alcohol units (12 g) per week**				
0	73	31	42	0.603††
< 1	36	13	23	
1-2	91	39	52	
≥ 3	18	10	8	
**Food allergies**				
No	210	89	121	0.669††
Yes	8	4	4	

†p.value calculated via Welch's t-test; ††p.value calculated via chi-squared test.
*mean ± SD; **n

Table S3: Demographic (mean ± SD), lifestyle (n), and diet-related (n) characteristics of participants (n = 218) whose FFQ data passed the screening for misreporting and outliers [11,12]. Differences between girls (n = 97) and boys (n = 125) are also listed (p.value). Values in bold are intended as statistically significant ($p < 0.05$). IPAQ: International Physical Activity Questionnaire [10].

Gj				Cp			
**Predictors**	**VIF**	**DW**	**p.value**	**Predictors**	**VIF**	**DW**	**p.value**
Σ sweet	1.643			Σ sweet	1.957		
Gender [G]	1.023	2.173	0.905	Gender [G]	1.009	2.227	0.957
Σ sweet × Gender [G]	1.640			Σ sweet × Gender [G]	1.951		
Σ sour	1.523			Σ bitter	1.502		
Gender [G]	1.001	2.203	0.939	Gender [G]	1.015	2.242	0.966
Σ sour × Gender [G]	1.523			Σ bitter × Gender [G]	1.502		
Σ bitter	1.858			Σ astringent	1.539		
Gender [G]	1.000	2.228	0.959	Gender [G]	1.000	2.270	0.979
Σ bitter × Gender [G]	1.858			Σ astringent × Gender [G]	1.539		
n		231		n		227	

Table S4: Variance Inflation Factor (VIF) and results from the Durbin-Watson test (DW) for the unadjusted general linear models assessing whether the relationship between global taste scores (Σ) and depressive symptoms varies by gender. Gj: grapefruit juice; Cp: dark chocolate pudding; Gender [G]: girls.

Gj				Cp			
**Predictors**	**VIF**	**DW**	**p.value**	**Predictors**	**VIF**	**DW**	**p.value**
Σ sweet	1.657			Σ sweet	1.974		
Gender [G]	1.182			Gender [G]	1.178		
SCARED	1.871			SCARED	1.857		
BIG-5 [N]	1.753	2.128	0.833	BIG-5 [N]	1.748	2.167	0.894
APEQ	1.202			APEQ	1.235		
BIDA	1.067			BIDA	1.064		
Σ sweet × Gender [G]	1.652			Σ sweet × Gender [G]	1.960		
Σ sour	1.542			Σ bitter	1.509		
Gender [G]	1.169			Gender [G]	1.177		
SCARED	1.865			SCARED	1.856		
BIG-5 [N]	1.752	2.144	0.862	BIG-5 [N]	1.747	2.147	0.863
APEQ	1.233			APEQ	1.230		
BIDA	1.068			BIDA	1.070		
Σ sour × Gender [G]	1.554			Σ bitter × Gender [G]	1.522		
Σ bitter	1.937			Σ astringent	1.596		
Gender [G]	1.160			Gender [G]	1.163		
SCARED	1.884			SCARED	1.868		
BIG-5 [N]	1.803	2.165	0.894	BIG-5 [N]	1.757	2.183	0.914
APEQ	1.254			APEQ	1.249		
BIDA	1.072			BIDA	1.073		
Σ bitter × Gender [G]	1.918			Σ astringent × Gender [G]	1.581		
n		231		n		227	

Table S5: Variance Inflation Factor (VIF) and results from the Durbin-Watson test (DW) for the adjusted general linear models assessing whether the relationship between global scores of sensory responsiveness (Σ) and depressive symptoms varies by gender. Gj: grapefruit juice; Cp: dark chocolate pudding; Gender [G]: girls. SCARED: generalized anxiety; BIG-5 [N]: neuroticism; APEQ: picky eating; BIDA: body dissatisfaction.

Product	Response	Predictors	Girls					Boys				
			β	CI 95 %	p.value	sr^2	R^2_{adj}	β	CI 95 %	p.value	sr^2	R^2_{adj}
Gj	Vitamin B6	Σ bitter	-0.221	-0.417 - -0.014	**0.035**	0.046	0.096	-0.017	-0.193 - 0.157	0.852	0.000	0.050
		SCARED	-0.076	-0.275 - 0.123	0.455	0.006		-0.064	-0.241 - 0.109	0.459	0.004	
		Age	0.109	-0.102 - 0.318	0.295	0.010		0.175	-0.005 - 0.351	0.055	0.029	
		BMI	-0.004	-0.197 - 0.201	0.961	0.000		0.137	-0.037 - 0.313	0.116	0.018	
		IPAQ	0.069	-0.124 - 0.274	0.487	0.005		0.123	-0.049 - 0.301	0.161	0.015	
		Σ bitter × SCARED	-0.251	-0.455 - -0.046	**0.016**	0.056		-0.173	-0.353 - 0.005	0.055	0.028	
	Potassium	Σ bitter	-0.158	-0.371 - 0.050	0.140	0.024	0.001	-0.077	-0.246 - 0.092	0.376	0.006	0.161
		SCARED	-0.14	-0.348 - 0.063	0.174	0.020		-0.013	-0.186 - 0.160	0.874	0.000	
		Age	0.011	-0.204 - 0.227	0.918	0.000		0.117	-0.052 - 0.291	0.171	0.013	
		BMI	0.030	-0.174 - 0.236	0.765	0.001		0.217	0.047 - 0.389	**0.012**	0.045	
		IPAQ	-0.05	-0.254 - 0.164	0.674	0.002		0.061	-0.107 - 0.231	0.492	0.004	
		Σ bitter × SCARED	-0.14	-0.354 - 0.069	0.186	0.018		-0.244	-0.422 - -0.074	**0.008**	0.055	
Cp	Fibers	Σ bitter	-0.300	-0.489 - -0.104	**0.004**	0.088	0.135	-0.011	-0.190 - 0.171	0.916	0.000	-0.014
		SCARED	0.000	-0.191 - 0.192	0.982	0.000		-0.095	-0.281 - 0.095	0.325	0.008	
		Age	-0.161	-0.361 - 0.041	0.122	0.024		0.098	-0.085 - 0.277	0.287	0.009	
		BMI	-0.053	-0.242 - 0.145	0.571	0.003		0.137	-0.049 - 0.321	0.149	0.017	
		IPAQ	-0.145	-0.336 - 0.047	0.140	0.021		0.036	-0.141 - 0.220	0.692	0.001	
		Σ bitter × SCARED	-0.273	-0.459 - -0.088	**0.005**	0.078		-0.018	-0.198 - 0.169	0.865	0.000	

Table S6: Moderating effects of generalized anxiety (SCARED) on the link between global responsiveness to alarming oral sensations (Σ) and habitual nutrient intake (Response), as a function of gender. Each model was adjusted for age, BMI, and level of physical activity (IPAQ). Bootstrapped β estimates and 95 % confidence intervals, along with p.values, sr^2, and adjusted R^2 are listed. Statistically significant main and interaction effects (p.value) are highlighted in bold. Gj: grapefruit juice; Cp: dark chocolate pudding.

Product	Response	Predictors	Girls					Boys				
			β	CI 95 %	p.value	sr^2	R^2_{adj}	β	CI 95 %	p.value	sr^2	R^2_{adj}
Gj	Proteins	Σ bitter	-0.125	-0.316 - 0.076	0.203	0.015	0.130	-0.146	-0.325 - 0.034	0.104	0.020	0.056
		BIG-5 [N]	-0.203	-0.394 - -0.012	**0.038**	0.041		0.032	-0.149 - 0.208	0.729	0.001	
		Age	-0.056	-0.268 - 0.146	0.593	0.003		0.071	-0.107 - 0.250	0.425	0.005	
		BMI	0.049	-0.139 - 0.252	0.626	0.002		0.052	-0.120 - 0.228	0.541	0.003	
		IPAQ	0.282	0.098 - 0.480	**0.003**	0.078		0.248	0.079 - 0.432	**0.004**	0.060	
		Σ bitter × BIG-5 [N]	-0.238	-0.458 - -0.014	**0.038**	0.040		-0.108	-0.299 - 0.082	0.252	0.010	
Cp	Fibers	Σ bitter	-0.316	-0.509 - -0.114	**0.003**	0.095	0.112	0.022	-0.164 - 0.212	0.809	0.000	-0.009
		BIG-5 [N]	-0.035	-0.237 - 0.156	0.704	0.001		-0.035	-0.215 - 0.146	0.710	0.001	
		Age	-0.134	-0.333 - 0.066	0.184	0.017		0.118	-0.068 - 0.302	0.205	0.013	
		BMI	-0.043	-0.239 - 0.162	0.651	0.002		0.108	-0.081 - 0.295	0.260	0.011	
		IPAQ	-0.143	-0.337 - 0.051	0.152	0.020		0.038	-0.136 - 0.224	0.681	0.001	
		Σ bitter × BIG-5 [N]	-0.204	-0.371 - -0.040	**0.016**	0.057		0.126	-0.078 - 0.331	0.214	0.012	
	Calcium	Σ bitter	-0.114	-0.307 - 0.074	0.231	0.013	0.177	-0.133	-0.319 - 0.048	0.158	0.016	0.024
		BIG-5 [N]	-0.210	-0.402 - -0.018	**0.032**	0.043		-0.007	-0.188 - 0.169	0.932	0.000	
		Age	-0.260	-0.450 - -0.069	**0.007**	0.065		-0.085	-0.266 - 0.097	0.354	0.007	
		BMI	-0.114	-0.303 - 0.073	0.237	0.013		0.092	-0.093 - 0.278	0.329	0.008	
		IPAQ	0.239	0.050 - 0.424	**0.013**	0.057		0.223	0.052 - 0.400	**0.012**	0.050	
		Σ bitter × BIG-5 [N]	-0.237	-0.398 - -0.078	**0.004**	0.077		-0.079	-0.281 - 0.118	0.438	0.005	
	Phosphorus	Σ bitter	-0.180	-0.377 - 0.016	0.071	0.031	0.129	-0.170	-0.355 - 0.010	0.067	0.025	0.077
		BIG-5 [N]	-0.185	-0.381 - 0.009	0.061	0.033		0.010	-0.168 - 0.185	0.905	0.000	
		Age	-0.062	-0.257 - 0.140	0.541	0.004		0.042	-0.135 - 0.222	0.635	0.002	
		BMI	0.037	-0.156 - 0.232	0.689	0.001		0.130	-0.053 - 0.315	0.159	0.015	
		IPAQ	0.303	0.110 - 0.497	**0.002**	0.091		0.310	0.138 - 0.483	**0.001**	0.094	
		Σ bitter × BIG-5 [N]	-0.171	-0.336 - -0.006	**0.042**	0.040		-0.102	-0.299 - 0.097	0.310	0.008	
	Vitamin B1	Σ astringent	-0.323	-0.582 - -0.063	**0.017**	0.056	0.116	-0.022	-0.19 - 0.143	0.785	0.000	0.090
		BIG-5 [N]	-0.081	-0.292 - 0.117	0.426	0.006		-0.017	-0.184 - 0.148	0.850	0.000	
		Age	0.206	0.011 - 0.399	**0.037**	0.041		0.204	0.040 - 0.367	**0.016**	0.040	
		BMI	0.112	-0.082 - 0.311	0.267	0.012		0.207	0.041 - 0.377	**0.014**	0.040	
		IPAQ	0.110	-0.085 - 0.309	0.269	0.012		0.295	0.127 - 0.462	**< 0.001**	0.087	
		Σ astringent × BIG-5 [N]	0.024	-0.207 - 0.257	0.853	0.000		-0.237	-0.432 - -0.05	**0.014**	0.041	

Table S7: Moderating effects of neuroticism (BIG-5 [N]) on the link between global responsiveness to alarming oral sensations (Σ) and habitual nutrient intake (Response), as a function of gender. Each model was adjusted for age, BMI, and level of physical activity (IPAQ). Bootstrapped β estimates and 95 % confidence intervals, along with p.values, sr^2, and adjusted R^2 are listed. Statistically significant main and interaction effects (p.value) are highlighted in bold. Gj: grapefruit juice; Cp: dark chocolate pudding.

Product	Response	Predictors	Girls					Boys				
			β	CI 95 %	p.value	sr^2	R^2_{adj}	β	CI 95 %	p.value	sr^2	R^2_{adj}
Gj	Proteins	Σ sour	0.088	-0.125 - 0.303	0.419	0.007	0.059	-0.197	-0.367 - -0.022	**0.028**	0.037	0.083
		APEQ	-0.132	-0.359 - 0.103	0.262	0.013		0.046	-0.126 - 0.215	0.617	0.002	
		Age	0.012	-0.199 - 0.219	0.919	0.000		0.082	-0.089 - 0.258	0.347	0.006	
		BMI	0.045	-0.150 - 0.254	0.676	0.002		0.039	-0.131 - 0.213	0.645	0.001	
		IPAQ	0.248	0.054 - 0.449	**0.012**	0.061		0.224	0.055 - 0.403	**0.009**	0.048	
		Σ sour × APEQ	-0.095	-0.286 - 0.104	0.335	0.010		0.195	0.003 - 0.385	**0.047**	0.031	
	Saturated fats	Σ bitter	0.048	-0.175 - 0.265	0.651	0.002	0.098	-0.095	-0.273 - 0.086	0.304	0.008	0.020
		APEQ	-0.170	-0.387 - 0.052	0.131	0.023		0.189	0.007 - 0.374	**0.041**	0.033	
		Age	-0.174	-0.383 - 0.032	0.100	0.027		-0.184	-0.363 - -0.001	**0.049**	0.032	
		BMI	-0.085	-0.283 - 0.116	0.393	0.007		0.015	-0.160 - 0.200	0.859	0.000	
		IPAQ	0.214	0.015 - 0.411	**0.033**	0.044		-0.079	-0.252 - 0.102	0.369	0.006	
		Σ bitter × APEQ	-0.300	-0.531 - -0.080	**0.008**	0.068		-0.079	-0.262 - 0.098	0.365	0.006	
Cp	Fibers	Σ bitter	-0.285	-0.479 - -0.083	**0.008**	0.079	0.107	-0.006	-0.184 - 0.173	0.955	0.000	0.004
		APEQ	0.105	-0.096 - 0.307	0.315	0.010		-0.165	-0.349 - 0.015	0.072	0.025	
		Age	-0.085	-0.282 - 0.119	0.389	0.007		0.121	-0.061 - 0.301	0.190	0.014	
		BMI	-0.036	-0.232 - 0.169	0.710	0.001		0.150	-0.035 - 0.334	0.110	0.020	
		IPAQ	-0.175	-0.371 - 0.025	0.082	0.029		0.039	-0.139 - 0.222	0.665	0.001	
		Σ bitter × APEQ	-0.249	-0.480 - -0.024	**0.030**	0.045		0.002	-0.208 - 0.215	0.978	0.000	
	Vitamin B2	Σ bitter	-0.068	-0.264 - 0.136	0.494	0.005	0.097	-0.021	-0.198 - 0.166	0.802	0.000	-0.011
		APEQ	0.130	-0.067 - 0.327	0.205	0.016		-0.132	-0.321 - 0.056	0.170	0.016	
		Age	0.211	0.011 - 0.413	**0.038**	0.043		0.079	-0.101 - 0.263	0.393	0.006	
		BMI	0.056	-0.137 - 0.259	0.579	0.003		0.121	-0.066 - 0.313	0.202	0.013	
		IPAQ	0.115	-0.082 - 0.320	0.269	0.013		0.094	-0.078 - 0.285	0.300	0.009	
		Σ bitter × APEQ	-0.299	-0.528 - -0.067	**0.013**	0.065		-0.053	-0.271 - 0.157	0.626	0.002	

Table S8: Moderating effects of picky eating (APEQ) on the link between global responsiveness to alarming oral sensations (Σ) and habitual nutrient intake (Response), as a function of gender. Each model was adjusted for age, BMI, and level of physical activity (IPAQ). Bootstrapped β estimates and 95 % confidence intervals, along with p.values, sr^2, and adjusted R^2 are listed. Statistically significant main and interaction effects (p.value) are highlighted in bold. Gj: grapefruit juice; Cp: dark chocolate pudding.

Product	Response	Predictors	Girls					Boys				
			β	CI 95%	p.value	sr^2	R^2_{adj}	β	CI 95%	p.value	sr^2	R^2_{adj}
Gj	Vitamin B6	Σ sour	-0.203	-0.397 - -0.015	**0.033**	0.040	0.155	-0.079	-0.251 - 0.099	0.099	0.006	0.036
		BIDA	0.219	0.018 - 0.419	**0.032**	0.042		-0.118	-0.307 - 0.073	0.073	0.011	
		Age	0.181	-0.016 - 0.373	0.077	0.031		0.191	0.013 - 0.371	0.371	0.035	
		BMI	0.036	-0.164 - 0.244	0.723	0.001		0.160	-0.022 - 0.343	0.343	0.022	
		IPAQ	0.127	-0.060 - 0.321	0.174	0.016		0.105	-0.070 - 0.284	0.284	0.010	
		Σ sour × BIDA	-0.355	-0.584 - -0.136	**0.003**	0.092		0.031	-0.209 - 0.273	0.273	0.001	
	Vitamin B3	Σ bitter	-0.160	-0.346 - 0.032	0.099	0.025	0.197	0.036	-0.146 - 0.215	0.215	0.001	0.017
		BIDA	0.065	-0.127 - 0.255	0.512	0.004		-0.076	-0.262 - 0.110	0.110	0.005	
		Age	0.300	0.113 - 0.488	**0.003**	0.088		0.207	0.031 - 0.389	0.389	0.041	
		BMI	0.135	-0.052 - 0.330	0.160	0.017		0.155	-0.033 - 0.342	0.342	0.020	
		IPAQ	0.191	0.014 - 0.374	**0.034**	0.036		0.064	-0.111 - 0.246	0.246	0.004	
		Σ bitter × BIDA	-0.303	-0.530 - -0.082	**0.009**	0.062		0.074	-0.120 - 0.267	0.267	0.005	
	Vitamin B6	Σ bitter	-0.259	-0.454 - -0.061	**0.012**	0.062	0.175	-0.018	-0.199 - 0.159	0.159	0.000	0.029
		BIDA	0.187	-0.011 - 0.388	0.065	0.031		-0.114	-0.297 - 0.075	0.075	0.011	
		Age	0.180	-0.018 - 0.377	0.074	0.030		0.197	0.018 - 0.375	0.375	0.037	
		BMI	-0.028	-0.226 - 0.179	0.798	0.001		0.144	-0.037 - 0.331	0.331	0.018	
		IPAQ	0.076	-0.113 - 0.275	0.428	0.005		0.103	-0.072 - 0.284	0.284	0.010	
		Σ bitter × BIDA	-0.392	-0.630 - -0.156	**0.002**	0.098		-0.018	-0.209 - 0.175	0.175	0.000	
Cp	Proteins	Σ bitter	-0.135	-0.348 - 0.078	0.197	0.016	0.086	-0.145	-0.324 - 0.033	0.111	0.020	0.052
		BIDA	-0.131	-0.359 - 0.104	0.269	0.013		-0.043	-0.239 - 0.153	0.645	0.001	
		Age	0.074	-0.127 - 0.273	0.464	0.005		0.110	-0.065 - 0.286	0.211	0.012	
		BMI	0.111	-0.088 - 0.323	0.285	0.011		0.091	-0.086 - 0.276	0.328	0.007	
		IPAQ	0.257	0.057 - 0.463	**0.010**	0.063		0.252	0.075 - 0.436	**0.003**	0.061	
		Σ bitter × BIDA	-0.309	-0.603 - -0.031	**0.028**	0.047		0.007	-0.174 - 0.196	0.925	0.000	
	Phosphorus	Σ bitter	-0.237	-0.441 - -0.035	**0.023**	0.051	0.125	-0.132	-0.311 - 0.045	0.139	0.017	0.077
		BIDA	-0.105	-0.328 - 0.116	0.352	0.008		-0.100	-0.299 - 0.096	0.309	0.008	
		Age	-0.021	-0.213 - 0.176	0.846	0.000		0.062	-0.112 - 0.239	0.490	0.004	
		BMI	0.021	-0.183 - 0.223	0.820	0.000		0.132	-0.049 - 0.32	0.152	0.015	
		IPAQ	0.281	0.083 - 0.476	**0.005**	0.077		0.289	0.115 - 0.468	**0.002**	0.079	
		Σ bitter × BIDA	-0.363	-0.633 - -0.088	**0.010**	0.065		0.031	-0.149 - 0.215	0.724	0.001	
	Vitamin B1	Σ bitter	-0.363	-0.557 - -0.171	**0.001**	0.117	0.202	-0.068	-0.236 - 0.104	0.439	0.004	0.126
		BIDA	0.099	-0.120 - 0.311	0.383	0.007		-0.055	-0.246 - 0.133	0.560	0.002	
		Age	0.214	0.028 - 0.405	**0.026**	0.045		0.219	0.052 - 0.386	**0.012**	0.047	
		BMI	0.032	-0.155 - 0.230	0.741	0.001		0.200	0.03 - 0.377	**0.021**	0.036	
		IPAQ	0.097	-0.091 - 0.287	0.314	0.009		0.302	0.131 - 0.475	**< 0.001**	0.088	
		Σ bitter × BIDA	-0.318	-0.589 - -0.055	**0.017**	0.050		0.027	-0.151 - 0.204	0.767	0.001	
	Vitamin B6	Σ bitter	-0.212	-0.424 - -0.008	**0.041**	0.040	0.109	-0.091	-0.267 - 0.083	0.315	0.008	0.057
		BIDA	0.071	-0.158 - 0.293	0.553	0.004		-0.108	-0.306 - 0.091	0.278	0.009	
		Age	0.189	-0.006 - 0.39	0.056	0.035		0.195	0.022 - 0.366	**0.031**	0.037	
		BMI	-0.018	-0.22 - 0.188	0.869	0.000		0.193	0.014 - 0.378	**0.033**	0.033	
		IPAQ	0.094	-0.100 - 0.299	0.340	0.009		0.111	-0.064 - 0.29	0.220	0.012	
		Σ bitter × BIDA	-0.348	-0.640 - -0.072	**0.013**	0.060		0.100	-0.082 - 0.29	0.277	0.009	
	Phosphorus	Σ astringent	-0.333	-0.533 - -0.136	**0.002**	0.099	0.167	-0.004	-0.187 - 0.175	0.954	0.000	0.064
		BIDA	-0.186	-0.425 - 0.056	0.134	0.021		-0.078	-0.284 - 0.126	0.444	0.004	
		Age	-0.022	-0.208 - 0.168	0.821	0.001		0.083	-0.094 - 0.262	0.365	0.007	
		BMI	0.043	-0.152 - 0.240	0.652	0.002		0.100	-0.088 - 0.292	0.292	0.008	
		IPAQ	0.328	0.137 - 0.518	**0.001**	0.106		0.281	0.104 - 0.46	**0.002**	0.074	
		Σ astringent × BIDA	-0.521	-0.898 - -0.141	**0.009**	0.069		-0.074	-0.238 - 0.096	0.375	0.006	

Product	Response	Predictors	Girls					Boys				
			β	CI 95%	p.value	sr^2	R^2_{adj}	β	CI 95%	p.value	sr^2	R^2_{adj}
Cp	Vitamin B2	Σ astringent	-0.173	-0.380 - 0.050	0.118	0.027	0.083	-0.042	-0.232 - 0.157	0.639	0.002	-0.023
		BIDA	-0.083	-0.333 - 0.174	0.517	0.004		-0.060	-0.269 - 0.158	0.549	0.003	
		Age	0.213	0.016 - 0.416	**0.033**	0.045		0.075	-0.110 - 0.264	0.422	0.005	
		BMI	0.035	-0.169 - 0.244	0.734	0.001		0.100	-0.097 - 0.300	0.316	0.008	
		IPAQ	0.171	-0.028 - 0.374	0.092	0.029		0.087	-0.091 - 0.283	0.360	0.007	
		Σ astringent × BIDA	-0.443	-0.843 - -0.045	**0.029**	0.050		-0.025	-0.197 - 0.151	0.753	0.001	

Table S9: Moderating effects of body dissatisfaction (BIDA) on the link between global responsiveness to alarming oral sensations (Σ) and habitual nutrient intake (Response), as a function of gender. Each model was adjusted for age, BMI, and level of physical activity (IPAQ). Bootstrapped β estimates and 95 % confidence intervals, along with p.values, sr^2, and adjusted R^2 are listed. Statistically significant main and interaction effects (p.value) are highlighted in bold. Gj: grapefruit juice; Cp: dark chocolate pudding.

References

1. Ervina, E.; Almli, V.L.; Berget, I.; Spinelli, S.; Sick, J.; Dinnella, C. Does Responsiveness to Basic Tastes Influence Preadolescents' Food Liking? Investigating Taste Responsiveness Segment on Bitter-Sour-Sweet and Salty-Umami Model Food Samples. *Nutrients* **2021**, *13*, 2721, doi:10.3390/nu13082721.

2. Monteleone, E.; Spinelli, S.; Dinnella, C.; Endrizzi, I.; Laureati, M.; Pagliarini, E.; Sinesio, F.; Gasperi, F.; Torri, L.; Aprea, E.; et al. Exploring Influences on Food Choice in a Large Population Sample: The Italian Taste Project. *Food Qual Prefer* **2017**, *59*, 123–140, doi:10.1016/j.foodqual.2017.02.013.

3. Hayes, J.E.; Allen, A.L.; Bennett, S.M. Direct Comparison of the Generalized Visual Analog Scale (GVAS) and General Labeled Magnitude Scale (GLMS). *Food Qual Prefer* **2013**, *28*, 36–44, doi:10.1016/j.foodqual.2012.07.012.

4. Menghi, L.; Endrizzi, I.; Cliceri, D.; Zampini, M.; Giacalone, D.; Gasperi, F. Validating the Italian Version of the Adult Picky Eating Questionnaire. *Food Qual Prefer* **2022**, *101*, doi:10.1016/j.foodqual.2022.104647.

5. He, J.; Ellis, J.M.; Zickgraf, H.F.; Fan, X. Translating, Modifying, and Validating the Adult Picky Eating Questionnaire for Use in China. *Eat Behav* **2019**, *33*, 78–84, doi:10.1016/j.eatbeh.2019.04.002.

6. Ellis, J.M.; Galloway, A.T.; Mary Webb, R.; Martz, D.M. Measuring Adult Picky Eating: The Development of a Multidimensional Self-Report Instrument. *Psychol Assess* **2017**, *29*, 955–966, doi:10.1037/pas0000387.

7. Kauer, J.; Pelchat, M.L.; Rozin, P.; Zickgraf, H.F. Adult Picky Eating. Phenomenology, Taste Sensitivity, and Psychological Correlates. *Appetite* **2015**, *90*, 219–228, doi:10.1016/j.appet.2015.03.001.

8. Sánchez-Miguel, P.A.; Vaquero-Solís, M.; Sánchez-Oliva, D.; Pulido-González, J.J.; Segura-García, C.; Tapia-Serrano, M.A. Validation of the Body Image Dimensional Assessment in Adolescents from Spanish High School. *Eating and Weight Disorders* **2021**, *26*, 1749–1756, doi:10.1007/S40519-020-00992-7.

9. Añez, E.; Fornieles-Deu, A.; Fauquet-Ars, J.; López-Guimerà, G.; Puntí-Vidal, J.; Sánchez-Carracedo, D. Body Image Dissatisfaction, Physical Activity and Screen-Time in Spanish Adolescents. *J Health Psychol* **2018**, *23*, 36–47, doi:10.1177/1359105316664134.

10. Mannocci, A.; Di Thiene, D.; Del Cimmuto, A.; Masala, D.; Boccia, A.; De Vito, E. International Physical Activity Questionnaire: Validation and Assessment in an Italian Sample. *Italian Journal of Public Health* **2010**, *7*.

11. Welch, A.A.; Luben, R.; Khaw, K.T.; Bingham, S.A. The CAFE Computer Program for Nutritional Analysis of the EPIC-Norfolk Food Frequency Questionnaire and Identification of Extreme Nutrient Values. *Journal of Human Nutrition and Dietetics* **2005**, *18*, 99–116, doi:10.1111/j.1365-277X.2005.00593.x.

12. Mazzeo, T.; Roncoroni, L.; Lombardo, V.; Tomba, C.; Elli, L.; Sieri, S.; Grioni, S.; Bardella, M.T.; Agostoni, C.; Doneda, L.; et al. Evaluation of a Modified Italian European Prospective Investigation into Cancer and Nutrition Food Frequency Questionnaire for Individuals with Celiac Disease. *J Acad Nutr Diet* **2016**, *116*, 1810–1816, doi:10.1016/j.jand.2016.04.013.
